# Association of electrolytes and complete blood count in adolescent depression with and without psychotic symptoms

**DOI:** 10.1186/s12888-025-06906-0

**Published:** 2025-05-07

**Authors:** Xinyuan Li, Ziming Liu, Yanming Li, Xiuyu Jin, Shumin Zhu, Zining Liu, Xintong Pang, Yulan Geng

**Affiliations:** 1 Department of Laboratory Medicine, Hebei Children’s Hospital, Shijiazhuang, China; 2 Computer Network Information Center, Chinese Academy of Sciences, BeiJing, China; 3 Department of Laboratory Medicine, The First Hospital of Hebei Medical University, 89 Donggang Road, Shijiazhuang, 050031 China; 4 Department of Laboratory Medicine, Hebei Medical University Third Hospital, Shijiazhuang, China

**Keywords:** Depression, Electrolytes, Complete blood count, Adolescents, Psychiatric symptom

## Abstract

**Background:**

The aim of this study was to investigate physiological differences in electrolytes and complete blood counts in adolescent patients with depression with and without psychotic symptoms. By comparing baseline data in adolescent patients, it will provide more comprehensive information for individualised diagnosis and treatment of depression.

**Methods:**

Clinical baseline data of adolescent patients were collected, including information on gender, age, smoking history, and alcohol consumption history. In terms of electrolytes and complete blood counts, the differences between the two groups of patients were compared, and a predictive model was constructed by stepwise logistic regression, and its diagnostic value was evaluated by ROC.

**Results:**

Ca, WBC and NE were relevant factors for the development of psychotic symptoms in adolescents (Ca: OR = 21.95; WBC: OR = 1.16; NE: OR = 1.18). The three indicators and the constructed predictive model 1 performed poorly in the ROC curve in adolescent patients, with an AUC of 0.598.

**Conclusion:**

Blood calcium plays an important role in adolescent depression with psychotic symptoms. And leukocytes, neutrophils in depression with psychotic symptoms as an indicator of inflammation suggestive indicators for treatment and mechanism studies.

**Limitations:**

This study was a cross-sectional study. The study population was Chinese adolescents and did not include adolescents from other regions.

**Clinical trial number:**

Not applicable.

## Highlights

Psychotic Depression Biomarkers: Elevated serum calcium (Ca), white blood cell (WBC), and neutrophil (NE) levels were identified as significant relevant factors for psychotic depression (PD) in adolescents, providing potential biomarkers for early diagnosis.

Predictive Model Development: A logistic regression model incorporating Ca, WBC, and NE showed some predictive value for psychotic symptoms, though with limited accuracy, indicating room for refinement.

Recommendations for Future Research: Longitudinal studies, integration of multi-modal data (e.g., biomarkers, neuroimaging, and genomics), and exploration of immune cell subpopulations are suggested to deepen understanding of PD mechanisms.

## Introduction

Psychotic Depression (PD) is a subtype of depression characterized by the presence of psychotic symptoms, such as hallucinations, delusions, and anxiety. These symptoms occur during depressive episodes and typically subside as the depression resolves [1, 2]. Compared to non-psychotic depression, PD presents with more severe symptoms, a higher relapse rate, and a poorer prognosis [3]. Epidemiological evidence suggests that approximately 0.35-1% of the general population will experience PD at some point in their lives [4].Additionally, patients with PD have worse clinical outcomes and a higher mortality rate compared to those with Non-Psychotic Depression (NPD) [5].In recent years, researchers have increasingly focused on the physiological mechanisms underlying PD, particularly the role of electrolyte imbalances and abnormalities in complete blood count (CBC).

Studies have suggested that blood biochemical markers may be associated with the pathophysiology of depression. For instance, research on adult patients with depression has indicated that elevated levels of calcium (Ca) and sodium (Na) may be linked to the severity of depressive symptoms [6]. Additionally, the role of inflammatory responses in the pathogenesis of depression has gained significant attention. Some studies have found that increased levels of white blood cells (WBC) and neutrophils (NE) may be related to the inflammatory state in depressed individuals, particularly those with psychotic symptoms [7]. However, systematic research on electrolyte and CBC markers in adolescent patients with depression, especially those with psychotic features, remains limited.

Building upon this background, the present study proposes the hypothesis that blood electrolytes and CBC parameters exhibit significant abnormalities in adolescents with depression accompanied by psychotic symptoms and may be associated with the onset of these symptoms.To validate this hypothesis, this study conducts a comparative analysis of electrolyte and CBC markers between adolescents with depression with and without psychotic symptoms.

## Methods

### Study design

This study is a retrospective cross-sectional study, with data collected through the hospital medical record system. The study population consists of adolescent inpatients diagnosed with major depressive episodes, with or without psychotic symptoms, at The First Hospital of Hebei Medical University between May and December 2023.This study was reviewed by the Ethics Committee of the First Hospital of Hebei Medical University under the ethical number Research and Review 2,024,021.

### Diagnostic criteria

Diagnosis was based on the 10th edition of the International Classification of Diseases (ICD-10). The included participants met the criteria for the following ICD-10 codes: F32 for Major Depressive Episode, F33 for Recurrent Depressive Disorder, and F32.3/F33.3 for Severe Depressive Episode with Psychotic Symptoms.

### Inclusion and exclusion criteria

Participants were included if they were adolescents aged 12–18 years diagnosed with depression according to ICD-10 (F32, F33, F32.3, F33.3) and had completed electrolyte and CBC testing during hospitalization. Patients were excluded if they had other severe psychiatric disorders such as schizophrenia or bipolar disorder, had recently used medications affecting electrolyte levels, or had a history or presence of severe physical illnesses such as chronic kidney disease or malignant tumors. This study included both primary depressive cases (F32) and recurrent depressive disorder cases (F33), with statistical analysis distinguishing between these groups based on prior psychiatric medication use.

### Data completeness and handling of missing values

Among the collected laboratory data, two cases lacked CBC data and six cases lacked electrolyte data. Missing values were treated as blank data without imputation.

### Demographic covariates and measurement tools

Demographic variables included sex (biological sex; social gender was not collected), age, family history of psychiatric disorders, body mass index (BMI), diabetes, hypertension, hyperlipidemia, thyroid disease, history of infection, inflammatory status, smoking history, alcohol consumption, psychiatric medication use, self-harm or suicidal behavior, and parental divorce. All demographic data were retrieved from the hospital medical record system, with disease classification based on ICD-10.

### Laboratory data collection

Laboratory data were obtained from routine blood tests conducted upon hospital admission. The collected parameters included electrolytes: potassium (K), sodium chloride (Cl), calcium, phosphate (P), and magnesium (Mg). Serum proteins included albumin (ALB), globulin (GLB), and the albumin-to-globulin ratio (ALB/GLB). Complete blood count (CBC) parameters comprised white blood cell count, neutrophil percentage (NEP), lymphocyte percentage (LYP), monocyte percentage (MOP), absolute neutrophil count, absolute lymphocyte count (LY), absolute monocyte count (MO), red blood cell count (RBC), hemoglobin (HGB), and platelet count (PLT). Additionally, inflammatory markers derived from CBC included platelet-to-lymphocyte ratio (PLR), platelet-to-white blood cell ratio (PWR), neutrophil-to-lymphocyte ratio (NLR), derived neutrophil-to-lymphocyte ratio (dNLR), lymphocyte-to-monocyte ratio (LMR), monocyte-to-lymphocyte ratio (MLR), systemic immune-inflammation index (SII), and systemic inflammatory response index (SIRI).

The inflammatory markers were calculated using the following formulas: PLR = PLT/LY, PWR = PLT/WBC, NLR = NE/LY, dNLR = NE/(WBC - NE), LMR = LY/MO, MLR = MO/LY, SII = PLT × NE/LY, and SIRI = NE × MO/LY.

### Statistical analysis

All statistical analyses were performed using Free Statistics software (version 1.9) and R software (R Foundation for Statistical Computing, Vienna, Austria). Continuous variables following a normal distribution were expressed as mean ± standard error (SE), while non-normally distributed continuous variables were expressed as median (interquartile range, IQR). Categorical variables were expressed as numbers and percentages. Comparisons between groups were conducted using Student’s t-test for normally distributed continuous variables, the Mann-Whitney U-test for non-normally distributed continuous variables, and the chi-square test or Fisher’s exact test for categorical variables.

Binary logistic regression models were used to determine adjusted odds ratios (ORs) and 95% confidence intervals (CIs) for the associations between biochemical markers, CBC parameters, and the presence of psychotic symptoms. Although other demographic confounders were considered, due to data limitations, only inflammatory status showed statistical significance, while other demographic variables did not.Trend p-values were calculated using linear regression models for continuous variables and Wilcoxon rank-sum tests for categorical variables. Subgroup analysis based on sex and age (age < 15 vs. ≥15 years) was performed using multivariate logistic regression models. Stepwise logistic regression was applied to develop a predictive model. The formula, 7.188 + 0.117WBC + 0.002NE + 2.471*Ca, was derived through stepwise selection of significant predictors in a logistic regression model. The coefficients were obtained by fitting the model to the data, optimizing for the best discrimination between cases with and without psychotic symptoms. Receiver Operating Characteristic (ROC) curve analysis was conducted to evaluate its diagnostic performance.

## Results

### Clinical baseline data on 357 patients in adolescents

We analysed clinical baseline data on 357 adolescents with depression. The results showed that there were no significant differences in BMI, prevalence of chronic diseases, family history, thyroid disorders and psycho-behavioural factors among patients with or without psychiatric symptoms. However, there was a significant difference in the relatively high incidence of inflammation in the PD group (*P* < 0.05, Table 1). Among the electrolyte indices, there was a difference in Ca (*P* < 0.05, Table 1). Among them, PD patients showed higher levels of Ca compared to NPD patients. In terms of complete blood count, there was no significant difference between the two groups of patients in most of the indices (*P* > 0.05, Table 1). However, there were significant differences in WBC and NE, where depressed patients with psychotic symptoms showed higher levels (*P* < 0.05, Table 1).

**Table 1:** Clinical baseline data for the adolescent PD group versus the NPD group

Project	Total(*n* = 357)	NPD(*n* = 227)	PD(*n* = 130)	*P*
Sex				0.53
male	92(25.8)	56(24.7)	36(27.7)	
female	265(74.2)	171(75.3)	94(72.3)	
Age	15.0(13.0, 16.0)	14.0(13.0, 16.0)	15.0(13.0, 16.0)	0.465
BMI	20.2(18.2, 23.5)	20.0(18.1, 22.9)	21.0(18.4, 24.0)	0.182
Diabetes				0.366
no	354(99.7)	225(100)	129(99.2)	
yes	1(0.3)	0(0)	1(0.8)	
Hypertension				1
no	351(99.2)	222(99.1)	129(99.2)	
yes	3(0.8)	2(0.9)	1(0.8)	
Hyperlipidaemia				0.286
no	329(92.7)	206(91.6)	123(94.6)	
yes	26(7.3)	19(8.4)	7(5.4)	
Family history of mental illness				0.229
no	292(82.0)	182(80.2)	110(85.3)	
yes	64(18.0)	45(19.8)	19(14.7)	
Thyroid disease				0.652
no	236(66.1)	152(67)	84(64.6)	
yes	121(33.9)	75(33)	46(35.4)	
Whether or not an infection has occurred				0.106
no	346(96.9)	223(98.2)	123(94.6)	
yes	11(3.1)	4(1.8)	7(5.4)	
Whether there is inflammation				0.045
no	335(93.8)	218(96)	117(90)	
sinusitis	10(2.8)	3(1.3)	7(5.4)	
others	12(3.4)	6(2.6)	6(4.6)	
Have you ever taken psychotropic drugs?				0.654
no	59(16.5)	36(15.9)	23(17.7)	
yes	298(83.5)	191(84.1)	107(82.3)	
Any self-inflicted injuries or suicidal behaviour				0.324
no	97(27.2)	57(25.1)	40(30.8)	
self-injury	160(44.8)	101(44.5)	59(45.4)	
commit suicide	100(28.0)	69(30.4)	31(23.8)	
Smoking				0.163
no	350(98.3)	220(97.3)	130(100)	
yes	5(1.4)	5(2.2)	0(0)	
Drinking				0.267
no	348(97.8)	219(96.9)	129(99.2)	
yes	8(2.2)	7(3.1)	1(0.8)	
Whether parents are divorced				0.373
no	265(98.1)	165(98.8)	100(97.1)	
yes	5(1.9)	2(1.2)	3(2.9)	
K(mmol/L)	3.9 ± 0.3	3.9 ± 0.2	4.0 ± 0.3	0.223
Na(mmol/L)	139.0(137.6, 140.0)	138.2(137.0, 140.0)	139.0(138.0, 140.0)	0.496
CL(mmol/L)	104.7(103.5, 106.2)	104.7(103.5, 106.3)	104.6(103.6, 105.8)	0.459
Ca(mmol/L)	2.4(2.3, 2.4)	2.4(2.3, 2.4)	2.4(2.3, 2.5)	0.009
P(mmol/L)	1.4(1.3, 1.5)	1.4(1.3, 1.6)	1.4(1.3, 1.5)	0.979
MG(mmol/L)	0.9 ± 0.1	0.9 ± 0.1	0.9 ± 0.1	0.325
ALB(g/L)	42.6(40.5, 44.8)	42.5(40.4, 44.5)	43.0(40.6, 45.3)	0.133
GLB(g/L)	26.0 ± 3.5	26.0 ± 3.5	25.9 ± 3.5	0.71
ALB/GLB	1.6(1.5, 1.8)	1.6(1.5, 1.8)	1.7(1.5, 1.9)	0.172
WBC(10^9/L)	6.3 (5.3, 7.3)	6.2 (5.2, 7.0)	6.4 (5.5, 7.7)	0.028
NEP(%)	49.4 ± 10.1	48.9 ± 9.9	50.3 ± 10.4	0.206
LYP(%)	39.9 ± 9.6	40.3 ± 9.3	39.2 ± 10.1	0.285
MOP(%)	7.4 (6.3, 8.8)	7.4 (6.4, 9.1)	7.3 (6.1, 8.2)	0.133
NE(10^9/L)	3.0 (2.3, 4.0)	2.9 (2.3, 3.7)	3.1 (2.5, 4.3)	0.045
LY(10^9/L)	2.5 (2.0, 2.9)	2.5 (2.0, 2.9)	2.5 (2.0, 3.0)	0.589
MO(10^9/L)	0.5 (0.4, 0.6)	0.5 (0.4, 0.6)	0.5 (0.4, 0.6)	0.817
RBC(10^12/L)	4.3 (4.1, 4.7)	4.3 (4.0, 4.6)	4.3 (4.1, 4.7)	0.439
HGB(g/L)	127.0 (118.0, 137.5)	126.0 (117.0, 136.0)	129.0 (118.0, 138.0)	0.362
PLT(10^9/L)	253.0 (217.5, 289.0)	250.5 (216.0, 282.0)	255.0 (222.0, 305.0)	0.079
PLR	100.7 (83.2, 123.3)	100.0 (82.8, 120.1)	103.6 (85.0, 127.7)	0.458
PWR	16.1 (12.7, 21.4)	16.4 (12.2, 21.6)	15.4 (13.0, 20.9)	0.498
NLR	1.2 (0.9, 1.7)	1.2 (0.9, 1.6)	1.3 (0.9, 1.8)	0.187
dNLR	1.0 (0.8, 1.2)	0.9 (0.8, 1.2)	1.0 (0.8, 1.3)	0.195
LMR	5.2 (4.2, 6.6)	5.2 (4.0, 6.5)	5.2 (4.2, 6.8)	0.602
MLR	0.2 (0.2, 0.2)	0.2 (0.2, 0.2)	0.2 (0.1, 0.2)	0.602
SII	117.6 (80.7, 197.0)	113.8 (80.4, 187.4)	130.3 (81.7, 209.4)	0.183
SIRI	0.6 (0.4, 0.8)	0.6 (0.4, 0.8)	0.6 (0.4, 0.9)	0.257

Note: PD Psychotic Depression; NPD Non-Psychotic Depression; BMI: Body mass index; K: Potassium; Na: Sodium; Cl: Chloride; Ca: Calcium; P: Phosphate; Mg: Magnesium; ALB: Albumin; GLB: Globulin; ALB/GLB: Albumin-to-globulin ratio; WBC: White blood cell count; NEP: Neutrophil percentage; LYP: Lymphocyte percentage; MOP: Monocyte percentage; NE: Absolute neutrophil count; LY: Absolute lymphocyte count; MO: Absolute monocyte count; RBC: Red blood cell count; HGB: Hemoglobin; PLT: Platelet count; PLR: Platelet-to-lymphocyte ratio; PWR: Platelet-to-white blood cell ratio; NLR: Neutrophil-to-lymphocyte ratio; dNLR: Derived neutrophil-to-lymphocyte ratio; LMR: Lymphocyte-to-monocyte ratio; MLR: Monocyte-to-lymphocyte ratio; SII: Systemic immune-inflammation index; SIRI: Systemic inflammatory response index

### Logistic regression analysis of electrolyte and complete blood counts and their derived inflammatory markers in adolescent patients

In the logistic regression analysis, after adjusting for covariates, Ca, WBC, and NE levels were identified as possible associated factors for the occurrence of psychotic symptoms. The results showed that higher Ca levels were significantly related to an increased likelihood of psychotic symptoms (OR = 21.95, 95% CI: 2.06 ~ 233.57, *P* = 0.01), while elevated WBC (OR = 1.16, 95% CI: 1.01 ~ 1.32, *P* = 0.032) and NE levels (OR = 1.18, 95% CI: 1.00 ~ 1.38, *P* = 0.046) were also found to be possible influencing factors (Table 2).

**Table 2:** Logistic regression analysis of indicators related to the adolescent PD group versus the NPD group

Project	OR (95%CI)	*P*	OR (95%CI) ^a^	*P* ^a^
Ca(mmol/L)	17.13 (1.66 ~ 176.19)	0.017	21.94(2.06 ~ 233.57)	0.01
WBC(10^^^9/L)	1.16 (1.01 ~ 1.32)	0.031	1.16(1.01 ~ 1.32)	0.032
NE(10^^^9/L)	1.18 (1 ~ 1.38)	0.044	1.18(1 ~ 1.38)	0.046

Note: a indicates adjustment for sex and age in logistic regression analysis

PD Psychotic Depression; NPD Non-Psychotic Depression;*OR*: Odds Ratio; *CI*: Confidence Interval; Ca: Calcium; WBC: White blood cell count; NEP: Neutrophil percentage

### Subgroup analysis of Ca, WBC and NE indicators in adolescent patients

Logistic subgroup analyses of biochemical indices in adolescent depression patients revealed significant differences across subgroups (Table 3). Ca remained a possible relevant factor in females and patients aged ≥ 15 years, while WBC and NE were identified as relevant factors in patients aged ≥ 15 years.

**Table 3:** Logistic regression subgroup analysis of Ca, WBC and NE indicators in the adolescent PD group versus the NPD group

Project	group	OR(95CI)^a^	*P* ^a^	OR(95CI)^b^	*P* ^b^	*P* for interaction
Ca(mmol/L)						
	male	*22.13 (0.21 ~ 23*19.02)	0.192	25.91 (0.22 ~ 3090.39)	0.182	0.992
	female	13.95 (0.85 ~ 228.39)	0.065	17.81 (1.04 ~ 303.96)	0.047	
	age < 15	1.79 (0.07 ~ 44.43)	0.722	1.91 (0.08 ~ 48.73)	0.695	0.042
	age ≥ 15	168.13 (5.68 ~ 4978.09)	0.003	330.19 (9.91 ~ 10999.76)	0.001	
WBC(10^9/L)						
	male	1.13 (0.89 ~ 1.43)	0.324	1.16 (0.91 ~ 1.49)	0.23	0.949
	female	1.17 (1 ~ 1.37)	0.056	1.16 (0.99 ~ 1.36)	0.065	
	age < 15	1.08 (0.9 ~ 1.29)	0.407	1.08 (0.91 ~ 1.29)	0.381	0.297
	age ≥ 15	1.26 (1.03 ~ 1.54)	0.023	1.25 (1.02 ~ 1.53)	0.029	
NE(10^9/L)						
	male	1.12 (0.83 ~ 1.51)	0.457	1.12 (0.82 ~ 1.53)	0.478	0.748
	female	1.2 (0.99 ~ 1.46)	0.058	1.2 (0.99 ~ 1.45)	0.062	
	age < 15	1.07 (0.86 ~ 1.34)	0.516	1.08 (0.86 ~ 1.34)	0.508	0.252
	age ≥ 15	1.31 (1.03 ~ 1.67)	0.026	1.3 (1.02 ~ 1.66)	0.031	

Note: a indicates adjustment for sex and age in logistic regression analysis

b indicates adjustment for sex, age and “Whether there is inflammation” in logistic regression analysis

PD Psychotic Depression; NPD Non-Psychotic Depression;*OR*: Odds Ratio; *CI*: Confidence Interval; Ca: Calcium; WBC: White blood cell count; NEP: Neutrophil percentage

### ROC analysis of Ca, WBC and NE indicators in adolescent patients

In this study, the efficacy of various indicators in distinguishing patients with major depression with and without psychotic symptoms was evaluated using ROC curve analysis. For Ca, the AUC was 0.584 with a cut-off value of 2.395, demonstrating moderate specificity (0.689) but relatively low sensitivity (0.465). The AUCs for WBC and NE were 0.57 and 0.564, respectively, both showing moderate specificity (0.761 and 0.668) but relatively low sensitivity (0.395 and 0.465).

**Fig. 1:**
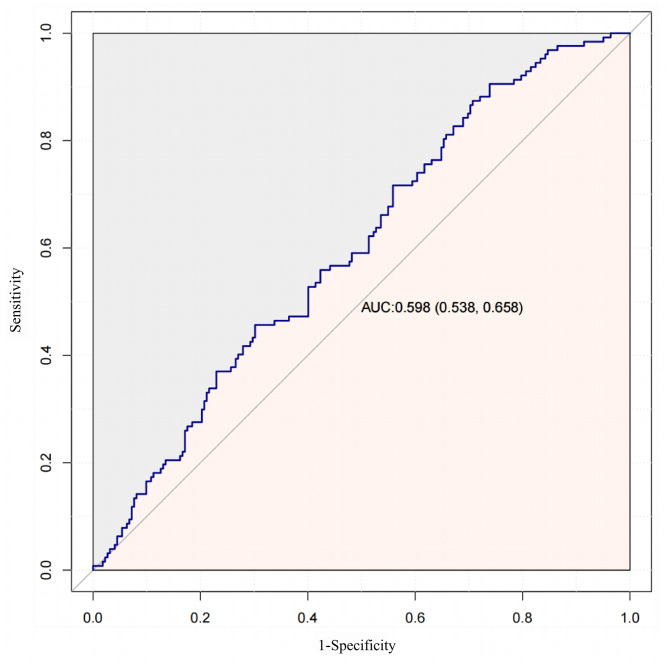
ROC analysis for model 1

To enhance predictive performance, stepwise logistic regression was used to combine these three indicators into a new predictive model: Model 1 = -7.188 + 0.117WBC + 0.002NE + 2.471*Ca. The AUC for Model 1 was 0.598, exhibiting high sensitivity (0.906) but low specificity (0.261) (Table 4; Fig. 1). These findings indicate that while these biochemical indicators provide some discriminatory value, their overall ability to differentiate depressive subtypes remains limited.

**Table 4:** Analysis of routine biochemical indices and complete blood counts and their derived inflammatory markers ROC in the adolescent PD and NPD groups

Project	AUC	95% CI	Cut-off Value	Specificity	Sensitivity
Ca(mmol/L)	0.584	0.522 ~ 0.647	2.395	0.689	0.465
WBC(10^9/L)	0.570	0.508 ~ 0.633	7.05	0.761	0.395
NE(10^9/L)	0.564	0.501 ~ 0.627	3.49	0.668	0.465
Modle 1	0.598	0.538 ~ 0.659	-0.827	0.261	0.906

Note: PD Psychotic Depression; NPD Non-Psychotic Depression; AUC Area Under the Curve; *CI* Confidence Interval; Ca: Calcium; WBC: White blood cell count; NEP: Neutrophil percentage

## Conclusion and directions for future research

PD is characterised by emotionally congruent hallucinations and/or delusions, and psychotic depression is often poorly diagnosed and treated [8]. Previous studies have highlighted the high probability of developing psychotic symptoms in patients with depression, with adolescents being particularly susceptible to such symptoms [9]. In this study, our findings suggest that Ca, WBC and NE are correlates of psychotic symptoms in adolescents.

In the present study, it was observed that in adolescents with depression accompanied by psychotic symptoms, there were high levels of Ca. Calcium in the blood exists in three forms—bound, complex, and free—with complex and free calcium constituting about half of the total [10]. Reduced calcium levels and neuronal calcium imbalances are linked to depressive symptoms. Calcium is essential for brain function, maintaining ionic homeostasis, supporting neurotransmitter regulation, and enhancing neuromuscular excitability. Studies have shown that calcium sensing in neurons is crucial for plasticity, neurotransmitter release, and synaptic modification, and that a deficiency in calcium sensing-1 leads to anxiety- and depression-like behaviors in mice [11 - 13]. Clinically, serum calcium and magnesium levels are correlated with the severity of depression, indicating that calcium plays a role in the condition, our findings support this, suggesting that elevated calcium levels contribute to more severe symptoms in PD compared to NPD [14].

WBC and NE showed significant differences in the PD group compared to NPD. This may be related to the activation of the immune system and inflammatory response. Previous studies have shown that inflammatory responses may play a key role in the development of depression [15]. Their identification as relevant factors for the development of psychotic symptoms in the present study emphasises the potential role of the immune system in depression. A randomised controlled study showed a significant increase in immune cell counts, especially neutrophils and monocytes, in depressed patients compared to controls, showing similarities to the results of the present study, i.e., an increase in immune cells versus neutrophils [16].Increases in WBCs and NEs may reflect activation of the immune system, which is consistent with the hypothesis of an inflammatory response in depression. Abnormal activity of the immune system may be involved in the pathophysiological process of depression by affecting the nervous system through multiple pathways. Taken together, there is a strong correlation between calcium indices and WBC and NE in complete blood counts and psychiatric symptoms in adolescent depressed patients. High calcium ion levels as well as activation of the immune system may be the biological mechanisms underlying this association.

Although this study provides valuable information for understanding electrolyte and complete blood counts in people with Parkinson’s disease, there are several limitations that may affect the interpretation of the findings. First, the sample size was relatively small and may not be fully representative of the broader Parkinson’s patient population.The wide confidence interval in the subgroup analysis may be due to the limited sample size, emphasizing the need for future studies to validate the findings with a larger sample size. In addition, the recruitment of hospitalized patients and specific selection criteria may introduce selection bias, thereby limiting the external validity of the results. Cross-sectional designs collect data at a single point in time, making it difficult to capture long-term changes in electrolytes and blood counts. Longitudinal studies can more effectively track dynamic changes in these metrics over the course of depression progression. Given that Parkinson’s disease is a multifactorial and multidimensional disorder, changes in electrolytes and blood cell counts may be influenced by factors such as inflammation, genetics, environment, and lifestyle. Although we collected a large amount of data, the study did not fully consider these interactions, which may limit conclusions.

To address these limitations, future research should adopt a longitudinal design to monitor the dynamics of electrolytes and blood counts over a longer period, particularly focusing on blood calcium levels and immune cell subpopulations to explore their role in PD development. Furthermore, integrating multi-modal data, including biomarkers, neuroimaging, and genomics, would provide deeper insights into the physiological mechanisms of PD, ultimately contributing to a more comprehensive understanding of this complex disorder.

## Data Availability

The datasets used and/or analyzed during the current study are available from the corresponding author on reasonable request.

## References

[1] Dubovsky SL, Ghosh BM, Serotte JC, Cranwell V. Psychotic depression: diagnosis, differential diagnosis, and treatment. Psychother Psychosom. 2021;90(3):160–77. 10.1159/000511348. 33166960 10.1159/000511348

[2] Yang R, Zhu F, Yue Y, Lu X, Zhu P, Li Z, Zhao X, Yang X, Zhou Y, Du X. Association between thyroid function and psychotic symptoms in adolescents with major depressive disorder: A large sample sized cross-sectional study in China. Heliyon. 2023;9(6):e16770. 10.1016/j.heliyon.2023.e16770. 37303557 10.1016/j.heliyon.2023.e16770 PMC10248252

[3] Paljärvi T, Tiihonen J, Lähteenvuo M, Tanskanen A, Fazel S, Taipale H. Mortality in psychotic depression: 18-year follow-up study. Br J Psychiatry: J Mental Sci. 2023;222(1):37–43. 10.1192/bjp.2022.140. 10.1192/bjp.2022.140 PMC10895511 36250518

[4] Gałuszko-Węgielnik M, Chmielewska Z, Jakuszkowiak-Wojten K, Wiglusz MS, Cubała WJ. Ketamine as Add-On treatment in psychotic treatment-Resistant depression. Brain Sci. 2023;13(1):142. 10.3390/brainsci13010142. 36672123 10.3390/brainsci13010142 PMC9856721

[5] Baird GS. Ionized calcium. Clinica chimica acta; international journal of clinical chemistry, 2011;412(9–10);696–701. 10.1016/j.cca.2011.01.004 10.1016/j.cca.2011.01.004 21238441

[6] Li X, Mao Y, Zhu S, Ma J, Gao S, Jin X, Wei Z, Geng Y. Relationship between depressive disorders and biochemical indicators in adult men and women. BMC Psychiatry. 2023;23(1):49. 10.1186/s12888-023-04536-y. 36653784 10.1186/s12888-023-04536-y PMC9847124

[7] Gaudiano BA, Young D, Chelminski I, Zimmerman M. Depressive symptom profiles and severity patterns in outpatients with psychotic vs nonpsychotic major depression. Compr Psychiatr. 2008;49(5):421–9. 10.1016/j.comppsych.2008.02.007. 10.1016/j.comppsych.2008.02.007 PMC2601715 18702928

[8] Le TT, Vincenzo D, Teopiz JD, Lee KM, Cha Y, Lui DS, Rodrigues LMW, Ho NB, Cao RC, Lin B, Nasri K, Gill F, Lipsitz H, Subramaniapillai O, Mansur M, Rosenblat RB, J. D., McIntyre RS. Ketamine for psychotic depression: an overview of the glutamatergic system and ketamine’s mechanisms associated with antidepressant and psychotomimetic effects. Psychiatry Res. 2021;306:114231. 10.1016/j.psychres.2021.114231. 34798487 10.1016/j.psychres.2021.114231

[9] Neufeld NH, Kaczkurkin AN, Sotiras A, Mulsant BH, Dickie EW, Flint AJ, Meyers BS, Alexopoulos GS, Rothschild AJ, Whyte EM, Mah L, Nierenberg J, Hoptman MJ, Davatzikos C, Satterthwaite TD, Voineskos AN. Structural brain networks in remitted psychotic depression. Neuropsychopharmacology: Official Publication Am Coll Neuropsychopharmacol. 2020;45(7):1223–31. 10.1038/s41386-020-0646-7. 10.1038/s41386-020-0646-7 PMC7235256 32109935

[10] Liu W, Wu Z, Sun M, Zhang S, Yuan J, Zhu D, Yan G, Hou K. Association between fasting blood glucose and thyroid stimulating hormones and suicidal tendency and disease severity in patients with major depressive disorder. Bosnian J Basic Med Sci. 2022;22(4):635–42. 10.17305/bjbms.2021.6754. 10.17305/bjbms.2021.6754 PMC9392982 35238287

[11] Jung KI, Ock SM, Chung JH, Song CH. Associations of serum Ca and Mg levels with mental health in adult women without psychiatric disorders. Biol Trace Elem Res. 2010;133(2):153–61. 10.1007/s12011-009-8421-y. 19543697 10.1007/s12011-009-8421-y

[12] Bach P, Schuster R, Koopmann A, Vollstaedt-Klein S, Spanagel R, Kiefer F. Plasma calcium concentration during detoxification predicts neural cue-reactivity and craving during early abstinence in alcohol-dependent patients. Eur Arch Psychiatry Clin NeuroSci. 2022;272(2):341–8. 10.1007/s00406-021-01240-4. 33630132 10.1007/s00406-021-01240-4 PMC8866328

[13] de Rezende VB, Rosa DV, Comim CM, Magno LA, Rodrigues AL, Vidigal P, Jeromin A, Quevedo J, Romano-Silva MA. NCS-1 deficiency causes anxiety and depressive-like behavior with impaired non-aversive memory in mice. Physiol Behav. 2014;130:91–8. 10.1016/j.physbeh.2014.03.005. 24631552 10.1016/j.physbeh.2014.03.005

[14] Linder J, Brismar K, Beck-Friis J, Sääf J, Wetterberg L. Calcium and magnesium concentrations in affective disorder: difference between plasma and serum in relation to symptoms. Acta Psychiatrica Scandinavica. 1989;80(6):527–37. 10.1111/j.1600-0447.1989.tb03021.x. 2618774 10.1111/j.1600-0447.1989.tb03021.x

[15] Rengasamy M, Da Costa E, Silva SA, Spada M, Price RB. Does the moderator matter? Identification of multiple moderators of the association between peripheral inflammatory markers and depression severity in a large Racially diverse community cohort. Neuropsychopharmacology: Official Publication Am Coll Neuropsychopharmacol. 2022;47(9):1693–701. 10.1038/s41386-022-01341-1. 10.1038/s41386-022-01341-1 PMC9283451 35595844

[16] Lynall ME, Turner L, Bhatti J, Cavanagh J, de Boer P, Mondelli V, Jones D, Drevets WC, Cowen P, Harrison NA, Pariante CM, Pointon L, Clatworthy MR, Bullmore E. & Neuroimmunology of mood disorders and Alzheimer’s disease (NIMA) consortium. Peripheral blood Cell-Stratified subgroups of inflamed depression. Biol Psychiatry, 2020;88(2):185–96. 10.1016/j.biopsych.2019.11.017 10.1016/j.biopsych.2019.11.017 32000983

